# *H1-MAPT* and the Risk for Familial Essential Tremor

**DOI:** 10.1371/journal.pone.0041581

**Published:** 2012-07-23

**Authors:** Elena García-Martín, Carmen Martínez, Hortensia Alonso-Navarro, Julián Benito-León, Oswaldo Lorenzo-Betancor, Pau Pastor, Tomás López-Alburquerque, Lluis Samaranch, Elena Lorenzo, José A. G. Agúndez, Félix Javier Jiménez-Jiménez

**Affiliations:** 1 Department of Biochemistry and Molecular Biology, Universidad de Extremadura, Cáceres, Spain; 2 Department of Pharmacology, Universidad de Extremadura, Badajoz, Spain; 3 Department of Medicine-Neurology, Hospital “Príncipe de Asturias” Universidad de Alcalá, Alcalá de Henares, Madrid, Spain; 4 Section of Neurology, Hospital Universitario del Sureste, Arganda del Rey, Madrid, Spain; 5 Service of Neurology, Hospital Doce de Octubre, Department of Medicine, Universidad Complutense, Madrid, Spain; 6 Centro de Investigación Biomédica en Red de Enfermedades Neurodegenerativas, Instituto de Salud Carlos III, Madrid, Spain; 7 Neurogenetics Laboratory, Division of Neurosciences, Center for Applied Medical Research, Universidad de Navarra, Pamplona, Spain; 8 Department of Neurology, Clínica Universidad de Navarra, University of Navarra School of Medicine, Pamplona, Spain; 9 Department of Neurology, Hospital Universitario de Salamanca, Salamanca, Spain; 10 Department of Pharmacology, Universidad de Extremadura, Cáceres, Spain; Centre Hospitalier Universitaire Vaudois (CHUV), Switzerland

## Abstract

The most frequent *MAPT* H1 haplotype is associated with the risk for developing progressive supranuclear palsy and other neurodegenerative diseases such as Parkinson’s disease. A recent report suggests that the *MAPT* H1 is associated with the risk for developing essential tremor. We wanted to confirm this association in a different population. We analyzed the distribution of allelic and genotype frequencies of rs1052553, which is an H1/H2 SNP, in 200 subjects with familial ET and 291 healthy controls. *rs1052553* genotype and allelic frequencies did not differ significantly between subjects with ET and controls and were unrelated with the age at onset of tremor or gender, and with the presence of head, voice, chin, and tongue tremor. Our study suggests that the MAPT H1 *rs1052553* is not associated with the risk for developing familial ET in the Spanish population.

## Introduction

Essential tremor (ET) is characterized by postural or kinetic 4–12 Hz tremor involving mainly the hands and forearms, although it can also affect the head, chin, voice, and other body regions. Family history of tremor in ET subjects ranges from 17.4% to 100%, and it is significantly more frequent than in a healthy population. Linkage studies identified three susceptibility loci for familial ET mapped at chromosomes 3q13, 2p24.1, and 6p23, but the responsible genes have not been clearly identified [1].

In recent years, it has been reported that mutations in the microtubule-associated protein tau gene (*MAPT*) cause frontotemporal dementia with parkinsonism linked to the chromosome 17 [2]. Single nucleotide polymorphisms (SNPs) across a chromosomal region expanding 1.3 Mb of the *MAPT* H1 haplotype have shown association with the risk for developing Parkinson’s disease (PD) [3]–[8], progressive supranuclear palsy [3], [9], [10], corticobasal degeneration [3], and multiple system atrophy [11].

There are many clinical, epidemiologic, genetic, neuroimaging and neuropathological data suggesting a relationship between ET and PD [12]–[14]. A recent study been suggested that the SNP *rs1052553*, which discriminates between *MAPT* H1 and H2 haplotypes, is associated with the risk for developing ET, with an odds-ratio of 1.32 (1.03–1.67) [11]. We attempted to replicate this finding in the Spanish population with ET compared with healthy controls.

## Methods

We studied 200 unselected and unrelated patients with diagnostic criteria for definite ET [15] (99 men,101 women, mean age 65.7±16.1, mean age at onset of ET 48.2±18.1 years), and 291 sex-matched controls (146 men, 145 women, mean age 46.5±12.6 years). The absence of previous neurological diseases, normality of thyroid function, and positive family history of ET (al least 1 first-degree relative affected) were obligatory requisites for inclusion. The patients were recruited from the Movement Disorders Units of 3 Hospitals. Controls were healthy unrelated gender-matched Caucasian Spanish individuals, most of them students or professors from the University of Extremadura, who did not have tremor or other movement disorders. Although our control group was not age-matched, age is not a determinant factor for polymorphisms of drug-metabolising enzymes [16], and the mean age of onset of ET did not differ significantly from the mean age of controls. According to the prevalence rates of ET in Spain [17], the percentage of controls with the risk genotypes that could develop ET in the lapse between the mean age of controls and the mean age of cases should not influence the results of the study.

**Table 1:** *MAPT rs1052553* genotypes and allelic variants of patients with essential tremor (ET) and healthy volunteers.

DATA FROM THE PRESENT STUDY				
	*ET PATIENTS (N = 200,* *400 alleles)*	*CONTROLS (N = 291, 582* *alleles)*	*OR (95% CI), p*	*Negative predictive value* *(95% CI)*
**GENOTYPES**				
AA	104 (52.0; 45.1–58.9)	158 (54.3; 48.6–60.0)	0.91 (0.62–1.33), 0.617	0.58 (0.53–0.63)
AG	75 (37.5; 30.8–44.2)	111 (38.1; 32.6–43.7)	0.98 (0.66–1.43), 0.885	0.59 (0.56–0.63)
GG	21 (10.5; 6.3–14.7)	22 (7.6; 4.5–10.6)	1.43 (0.73–2.81), 0.258	0.60 (0.59–0.62)
**ALLELES**				
A	283 (70.8; 66.3–75.2)	427 (73.4; 69.8–77.0)	0.88 (0.64–1.18), 0.368	0.60 (0.58–0.62)
G	117 (29.3; 24.8–33.7)	155 (26.6; 23.0–30.2)	1.13 (0.85–1.53), 0.368	0.57 (0.52–0.62)
**DATA FROM THE PRESENT STUDY COMBINED WITH VILARIÑO-GÜELL ET AL. STUDY ** [**11**]				
	***ET PATIENTS (N = 539,*** ***1078 alleles)***	***CONTROLS (N = 697,*** ***1394 alleles)***	***OR (95% CI), p***	***Negative predictive value*** ***(95% CI)***
**GENOTYPES**				
AA	325 (60.3; 56.2–64.4)	389 (55.8; 52.1–59.5)	1.20 (0.95–1.52), 0.113	0.59 (0.56–0.62)
AG	175 (32.5; 28.5–36.4)	259 (37.2; 33.6–40.7)	0.81 (0.64–1.04), 0.087	0.55 (0.53–0.57)
GG	39 (7.2; 5.0–9.4)	49 (7.0; 5.1–8.9)	1.03 (0.65–1.63), 0.889	0.56 (0.56–0.57)
**ALLELES**				
A	825 (76.5; 74.0–79.1)	1037 (74.4; 72.1–76.7)	1.12 (0.93–1.36), 0.221	0.59 (0.55–0.62)
G	253 (23.5; 20.9–26.0)	357 (25.6; 23.3–27.9)	0.89 (0.74–1.08), 0.221	0.56 (0.55–0.57)

The values in each cell represent: number (percentage; 95% confidence intervals).

**Table 2:** *MAPT rs1052553* genotypes and allelic variants of patients with essential tremor and healthy volunteers distributed by gender.

	*ET WOMEN (N = 101, 202 ALLELES)*	*CONTROL WOMEN (N = 146, 292* *ALLELES)*	*INTERGROUP COMPARISON VALUES* *OR (95%CI) P*	*ET MEN (N = 99,* *198 ALLELES)*	*CONTROL MEN (N = 145, 290 ALLELES)*	*INTERGROUP COMPARISON VALUES OR (95%CI) P*
**GENOTYPES**						
AA	57 (56.4; 46.8–66.1)	79 (54.1; 46.0–62.2)	1.10 (0.64–1.89), 0.718	47 (47.5; 37.6–57.3)	79 (54.5; 46.4–62.6)	0.76 (0.44–1.30), 0.283
AG	31 (30.7; 21.7–39.7)	57 (39.0; 31.1–47.0)	0.69 (0.39–1.23), 0.179	44 (44.4; 34.7–54.2)	54 (37.2; 29.4–45.1)	1.35 (0.78–2.35), 0.261
GG	13 (12.9; 6.3–19.4)	10 (6.8; 2.8–10.9)	2.01 (0.78–5.20), 0.110	8 (8.1; 2.7–13.4)	12 (8.3; 3.8–12.8)	0.97 (0.35–2.69), 0.957
**ALLELES**						
A	145 (71.8; 65.6–78.0)	215 (73.6; 68.6–78.7)	0.91 (0.60–1.39), 0.650	138 (69.7; 63.3–76.1)	212 (73.1; 68.0–78.2)	0.85 (0.56–1.29), 0.412
G	57 (28.2; 22.0–34.4)	77 (26.4; 21.3–31.4)	1.10 (0.72–1.67), 0.650	60 (30.3; 23.9–36.7)	78 (26.9; 21.8–32.0)	1.18 (0.78–1.80), 0.412

The values in each cell represent: number (percentage; 95% confidence intervals).

All the participants were included in the study after giving written informed consent. This study was approved by the Ethics Committee of the University Hospital “Príncipe de Asturias”, University of Alcalá, (Carretera de Alcalá Meco s/n, Alcalá de Henares E28805 Spain). The study was conducted according to the principles expressed in the declaration of Helsinki.

Genotyping for *rs1052553* allelic variant was performed in genomic DNA obtained from venous blood samples of participants using *TaqMan* Assays (C_7563736_10, Applied Biosciences Hispania, Alcobendas, Madrid, Spain) designed to detect the SNP *rs1052553*. Detection was carried out by qPCR in an Eppendorf realplex thermocycler. The amplification conditions were as follows: after a denaturation time of 10 min at 96°C, 45 cycles of 92°C 15 sec 60°C 90 sec were carried out and fluorescence was measured at the end of each cycle and at endpoint. All samples were determined in triplicate and genotypes were assigned both by gene identification software (RealPlex 2.0, Eppendorf, Madrid, Spain) and by analysis of the reference cycle number for each fluorescence curve, calculated by the use of the CalQPlex algorithm (Eppendorf, Madrid, Spain).

The intergroup comparison values were calculated by using the chi-square or Fisher tests when appropriate. The 95% confidence intervals were also calculated. We calculated the statistical power for the sample sizes in this study, in the Vilariño-Güell et al. study [11], and in the pooled data from both studies, which was determined from allele frequencies with a genetic model analyzing the frequency for carriers of the disease gene taking as reference value the OR reported in the original study (1.32) [11]. The respective values with P = 0.05 for one-tailed and two-tailed associations were, respectively 61.8% and 49.4% in the present study, 76.9% and 66.3%, in the original study [11], and 92.4% and 86.8% in the pooled analysis of the two studies.

The negative predictive value was calculated as d/r2 (d = number of control individuals with the risk factor absent; r2 = sum of ET patients and controls with the risk factor absent). The Hardy-Weinberg equilibrium was confirmed by means of Arlequin software Ver. 2.000.

## Results

The frequencies of *rs1052553* genotypes and allelic variants in ET patients did not differ significantly from those of healthy controls (Table 1), and were in Hardy-Weinberg’s equilibrium. An analysis of the pooled data of the present study with those of the Vilariño-Güell et al. [11] report, did not show significant differences between the frequencies of *rs1052553* genotypes and allelic variants in ET patients compared with controls (Table 1).

Allele and genotype frequencies of *rs1052553* were not influenced either by gender or by the age at onset of tremor (Table 2). Mean±SD age at onset of tremor did not differ among the ET *rs1052553AA*, *rs1052553AG* and *rs1052553GG* carriers (46.3±16.8; 49.4±19.4, and 54.7±17.7 years, respectively).

The respective frequencies of the *rs1052553GG* genotype and the *rs1052553G* allelic variant found in the ET patients with head (n = 45 [8.9%]; 95% CI, 0.6–17.2%; and [25.6%]; 95% CI, 16.5–34.6%), voice (n = 36 [11.1%]; 95% CI, 0.8–21.4%; and [29,2%], 95% CI, 18.7–39.75), tongue (n = 16 [12.5%]; 95% CI, 3.7–28.7; and [37.5%]; 95% CI, 20.7–54.3%), and chin tremor (n = 11 [0%] and [18.2%]; 95% CI, 2.1–34.3%) did not differ significantly from those found in the control group.

## Discussion

To date, neither linkage studies nor case-control association studies have been able to identify conclusively any gene responsible for ET. Some association studies suggested a possible relationship of the risk of developing ET with the methylentetrahydrofolate reductase, alpha-synuclein, CYP2C19, and CYP2C9/8 polymorphisms, whereas others did not find any association with alpha-synuclein, CYP2D6, alcohol-dehydrogenase 2 (ADH2), glutathione-transferase P1 (GSTP1) (revised in reference [1]), and paraoxonase 1 (PON-1) [18] polymorphisms. Our group reported association between the *Thr105Ile* polymorphism major allele (*rs511558538*) at the histamine-N-methyltransferase gene (*HNMT*) and the risk for developing ET [19], a finding that was not replicated by other groups [20]. Although a metaanalysis on *dopaminergic receptor D3* (*DRD3*) gene polymorphism suggested an association between this polymorphism and the risk for developing ET [21], another study involving a large series of ET patients and controls did not support this association [22]. In addition, most of the gamma-aminobutyric acid (GABA) receptor genes [23]–[26] or GABA transporter genes [26] are unrelated with the risk for developing ET.

While a genome-wide association study (GWAS) [27] found a strong statistical association between *Leucine rich repeat and Ig domain containing Nogo receptor interacting protein-1* gene *(LINGO1*) *rs9652490* and *rs11856808* SNPs and the risk for developing ET in an Icelandic population, the results of further studies were controversial, and the results of a metaanalysis showed no association of the *rs9652490G* allele, and a weak association of the *rs11856808T* allele with the risk for ET, although both variants showed a weak association with the risk for developing familial ET [28].

In the present study, we found no significant differences either in the frequencies of the *rs1052553* genotypes or in the frequencies of the allelic variants of this SNP in patients with familial ET when compared with healthy controls. In addition *rs1052553* was unrelated with age at onset of ET, and with the presence of head, voice, tongue or chin tremor.

The *rs1052553* variant allele frequency observed in control individuals in this study (26.6%) is almost identical to that reported by Vilariño-Güell et al. [11] (24.9%). However, we did not observe a decreased *rs1052553* allelic frequency among ET patients reported by Vilariño-Güell et al. [11], but a slight non-significant increase in the variant allele frequency (Table 1). While in the present series we only used familial cases, in the report by Vilariño-Güell et al. [11] the percentage of patients with familial and sporadic ET is not specified.

The results of the present study suggest that the *rs1052553* SNP is not related with the risk for developing familial ET. Despite the small sample size in the present study, it is of note that the analysis of the pooled data of this study with those of the report by Vilariño-Güell et al. [11], which includes a total of 539 ET patients and 697 controls, show lack of association between the *rs1052553* SNP and the risk for ET. However, this result should be interpreted with caution, since the pooled analysis could be unreliable when the status of the ET (familial or sporadic) in the original study is unknown.
